# Beyond Bone Loss: A Biology Perspective on Osteoporosis Pathogenesis, Multi-Omics Approaches, and Interconnected Mechanisms

**DOI:** 10.3390/biomedicines13061443

**Published:** 2025-06-12

**Authors:** Yixin Zhao, Jihan Wang, Lijuan Xu, Haofeng Xu, Yu Yan, Heping Zhao, Yuzhu Yan

**Affiliations:** 1 Clinical Laboratory of Honghui Hospital, Xi’an Jiaotong University, Xi’an 710054, China; 727042677@163.com (Y.Z.);; 2 Yan’an Medical College, Yan’an University, Yan’an 716000, China

**Keywords:** osteoporosis pathogenesis, bone remodeling, osteoimmunology, epigenetics, multi-omics

## Abstract

Osteoporosis is a systemic bone disorder characterized by decreased bone mass and deteriorated microarchitecture, leading to an increased risk of fractures. Recent studies have revealed that its pathogenesis involves complex biological processes beyond bone remodeling, including oxidative stress, chronic inflammation, cellular senescence, osteoimmunology, gut microbiota alterations, and epigenetic modifications. Oxidative stress disrupts bone homeostasis by promoting excessive free radical production and osteoclast activity. Chronic inflammation and the accumulation of senescent cells impair skeletal repair mechanisms. Advances in osteoimmunology have highlighted the critical role of immune–bone crosstalk in regulating bone resorption and formation. Moreover, the gut–bone axis, mediated by microbial metabolites, influences bone metabolism through immune and endocrine pathways. Epigenetic changes, such as DNA methylation and histone modification, contribute to gene–environment interactions, affecting disease progression. Multi-omics approaches (genomics, proteomics, and metabolomics) systematically identify molecular networks and comorbid links with diabetes/cardiovascular diseases, revealing pathological feedback loops that exacerbate bone loss. In conclusion, osteoporosis pathogenesis extends beyond bone remodeling to encompass systemic inflammation, immunometabolic dysregulation, and gut microbiota–host interactions. Future research should focus on integrating multi-omics biomarkers with targeted therapies to advance precision medicine strategies for osteoporosis prevention and treatment.

## 1. Introduction

Osteoporosis, a prevalent skeletal disorder characterized by reduced bone density and compromised architecture, significantly increases fracture risk and global disease burden [1]. When bone mineral density (BMD) falls 2.5 standard deviations below the mean for healthy young adults (T-score ≤ −2.5), osteoporosis remains underdiagnosed, with its true prevalence often being inferred from fracture rates [1]. Globally, osteoporotic fractures account for 0.83% of non-communicable disease burden, with notable prevalence in aging populations: 10.3% in U.S. adults ≥ 50 years and 33.49% in middle-aged and elderly Chinese adults [2,3]. In the UK, there is a similar burden of osteoporosis: the authors of an epidemiological study hypothesized that one in two women and one in five men aged over 50 years will suffer an osteoporotic fracture [4]. The authors of a meta-analysis concluded that the global prevalence of osteoporosis is approximately 18.3% in the age range of 15–105 years, where the prevalence rate in women is 23.1%, compared with 11.7% in men [5]. In a previous study, it was estimated that the number of people worldwide at high risk of osteoporotic fracture will double from 2010 to 2040. Asia has the highest proportion in the world, followed by Europe [6]. In addition, geographical location also has an impact on osteoporosis fractures: studies have shown that the probability of hip fractures is lower in low-latitude countries, possibly because of prolonged sunlight exposure [7].

Osteoporosis is inherently multifactorial, arising from complex interactions between genetic predisposition, environmental factors, hormonal changes, metabolic dysregulation, and cellular signaling pathways. This complexity necessitates holistic investigative approaches. Advancements in omics technologies are revolutionizing our understanding of osteoporosis. Genome-wide association studies (GWASs) allow for the identification of susceptibility genes, while transcriptomics, particularly single-cell approaches, reveals cellular heterogeneity and signaling pathways in bone remodeling. Proteomics uncovers key proteins linked to BMD, offering insights into diagnostic markers and therapeutic targets. Metabolomics explores metabolic shifts in calcium, phosphorus, and energy balance, providing potential biomarkers for early detection. Emerging fields such as epigenomics and microbiome research allow for the further elucidation of gene–environment interactions and gut–bone axis dynamics. Multi-omics integration is poised to propel osteoporosis management into the era of precision medicine, enabling early warning, precise typing, and targeted therapies. Therefore, the use of omics methods can provide a more comprehensive understanding, treatment, and prevention of osteoporosis. Genomics has confirmed the impact of variations in genes such as collagen type I alpha1 (*COLIA1*) and receptor-related protein 5 (*LRP5*) on bone health through candidate gene association studies. GWASs identify numerous risk loci related to osteoporosis. With RNA sequencing (RNA-seq), non-coding RNAseq, single-cell RNA sequencing (ScRNA-seq), and other proteomic research methods, not only can the key transcription factors of osteoporosis be identified; the differentially expressed genes of osteoporosis can also be identified to determine the etiology. Commonly used research methods in proteomics include TMT proteomics, liquid chromatography–tandem mass spectrometry (LC–MS/MS), Matrix-Assisted Laser Desorption/Ionization Time-of-Flight/Time-of-Flight Mass Spectrometry (MALDI-TOF/TOF), etc. Proteomics helps us to find osteogenic and osteoclastic markers, including Alkaline Phosphatase (ALP), osterix (OSX), the C-terminal telopeptide of type I X-linked collagen (CTX), etc. Meanwhile, the proteomic analysis of plasma, exosomes, and various body fluids allows one to more easily obtain specimens for the diagnosis of osteoporosis. Mass spectrometry analysis is a common research method in metabolomics that has revealed that osteoporosis is related to amino acid metabolism, lipid metabolism, energy metabolism, etc. Multi-omics analysis allows for the integration of the research results of multiple omics, which are then systematically analyzed. At the same time, Machine Learning can be used to analyze vast and complex datasets [8].

Osteoporosis frequently co-occurs with cardiovascular, respiratory, and other systemic diseases, highlighting its role in multicomorbidity networks [9]. Understanding these comorbidities is crucial to developing preventive strategies and therapeutic interventions and reducing the socioeconomic burden.

This review synthesizes the latest omics-driven insights into osteoporosis pathogenesis, explores its comorbidities, and identifies future research directions to advance clinical applications and personalized management.

## 2. Pathogenesis

Through osteogenic differentiation, osteoblasts synthesize extracellular matrix proteins and become osteocytes, comprising more than 90% of all bone cells, embedded in the mineralized bone matrix. Osteoclasts resorb the bone matrix by adhering to the bone [10]. Osteogenic differentiation is influenced by a variety of signaling pathways, including Wnt, bone morphogenetic proteins (BMPs), transforming growth factor-β (TGF-β), hedgehog, parathyroid hormone (PTH), fibroblast growth factors (FGFs), Notch, and others. The key transcription factors involved include Runt-related transcription factor 2 (*Runx2*), osterix (*Osx*), β-catenin, activating transcription factor 4 (*Atf4*), etc. [11]. Osteoclasts are differentiated from hematopoietic stem cells (HSCs), a process which is stimulated by monocyte/macrophage colony-stimulating factor (M-CSF) and the activation of the receptor activator of nuclear factor kappa B (RANK) with its ligand (RANKL). Osteoprotegerin (OPG) is a soluble receptor that competes with RANK to bind RANKL, thereby limiting osteoclastogenesis [12]. Osteoporosis is caused by a breakdown in the balance between bone resorption and bone formation [12]. Thus, the disruption of the cells, molecules, and signals involved in bone formation and resorption may lead to osteoporosis. Given that osteoporosis is a multifactorial disease, we summarized several pathologic mechanisms.

### 2.1. Endocrinology

Estrogen deficiency is recognized as the predominant etiological factor in postmenopausal osteoporosis. The expression of estrogen receptors (ERs), including the ERα and ERβ isoforms, in osteoblasts, osteoclasts, and osteocytes underscores the multifaceted role of estrogen in bone homeostasis. Estrogen signaling exerts anabolic effects on bone through diverse molecular pathways. Specifically, estrogen, by binding to Erα, can augment Wnt/β-catenin and BMP signaling to increase osteogenesis [13]. Additionally, estrogen extends the life span of osteoblasts by inhibiting apoptosis [14]. Schiavi et al. demonstrated that estrogen receptor inhibition significantly reduces fibronectin expression (FN1), impairing matrix production in the absence of mechanical stimulation [15].

Estrogen also modulates osteoclast activity. In estrogen-deficient murine models, elevated levels of pro-inflammatory cytokines, including tumor necrosis factor-α (TNF-α), interleukin-1 (IL-1), and interleukin-6 (IL-6), drive osteoclast maturation and bone resorption [16]. Estrogen suppresses the RANKL- and M-CSF-induced differentiation of myelomonocytic precursors into osteoclasts [17] and enhances osteoblast-derived TGF-β production, which promotes osteoclast apoptosis [18]. Osteocytes, which are mechanosensitive cells, exhibit altered responsiveness to mechanical stimuli under estrogen regulation. Estrogen modulates fluid flow-induced intracellular calcium oscillations in osteocytes, potentially influencing their differentiation [19]. Furthermore, osteocytes regulate osteoclast activity via RANKL production [20].

Estrogen glucocorticoids exhibit dose-dependent effects on bone metabolism. At physiological levels, glucocorticoids stimulate osteoblasts to secrete Wnt ligands (e.g., Wnt7b, Wnt10b, and Wnt9a), promoting the osteogenic differentiation of mesenchymal stem cells (MSCs) [21]. Osteoblast-specific glucocorticoid knockout models exhibit delayed calvarial bone development and impaired mineralization [22]. However, excessive glucocorticoid exposure upregulates peroxisome proliferator-activated receptor gamma receptor 2 (PPARγ2), shifting osteogenic differentiation toward adipogenesis [23]. Glucocorticoids inhibit Wnt16 expression in osteoblasts and attenuate the Wnt3a-mediated activation of β-catenin signaling [24,25]. They also upregulate sclerostin (Sost) expression in osteocytes, further suppressing the Wnt pathway [23]. Glucocorticoids enhance osteoclastogenesis by increasing M-CSF and RANKL expression while inhibiting OPG [26]. Paradoxically, they prolong osteoclast life span and induce osteocyte apoptosis [22]. Additionally, glucocorticoids stimulate osteocytes to produce Wnt antagonists, including sclerostin and Dickkopf-related protein 1 (DKK1) [23].

**Figure 1:**
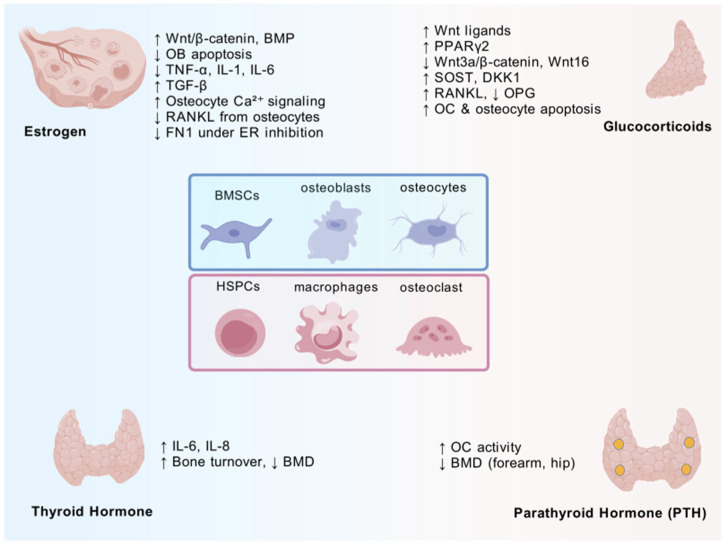
Hormonal regulation of bone cells contributing to osteoporosis. Estrogen deficiency, glucocorticoid excess, thyroid hormone excess, and elevated parathyroid hormone disrupt bone remodeling via cell-type-specific mechanisms. Key pathways and literature references are annotated. Created with biogdp.com [33].

Estrogen glucocorticoid hyperthyroidism and hyperparathyroidism are well-established causes of secondary osteoporosis. Thyroxine is critical to bone development and the maintenance of adult bone mass [27]. It accelerates bone turnover, shortening the remodeling cycle [28]. The authors of a two-sample Mendelian randomization analysis identified a causal relationship between hyperthyroidism and osteoporosis in European populations [29]. Thyroid hormone (T3) stimulates osteoblastic bone marrow stromal cells and osteoblast cell lines to produce IL-6 and IL-8, which promote osteoclastogenesis [30]. Despite advances in understanding the thyroid hormone’s role in bone turnover, its precise molecular mechanisms remain elusive. Hyperparathyroidism activates osteoclast activity, primarily affecting cortical bone, as evidenced by reduced BMD in the distal forearm and hip [31]. Trabecular bone may also be impacted, although the underlying mechanisms warrant further investigation [32]. These hormones change bone mass by influencing osteogenesis or osteogenesis (Figure 1).

### 2.2. Oxidative Stress and Inflammation

Oxidative stress and inflammation are central to the pathogenesis of osteoporosis, driving bone loss through their interplay and independent mechanisms. Oxidative stress, characterized by excessive reactive oxygen species (ROS) and reactive nitrogen species (RNS) [34], arises from mitochondrial dysfunction and cellular oxygen metabolism [35]. This process impairs bone formation by reducing osteoblastogenesis, increasing osteoblast and osteocyte apoptosis, and enhancing osteoclastogenesis [34]. Pathological conditions such as postmenopause [36], aging [37], hyperglycemia [38], and elevated fatty acids [39] exacerbate ROS accumulation, further compromising bone health.

Hydrogen peroxide (H_2_O_2_), a model for oxidative stress, inhibits the osteogenic differentiation of bone marrow stromal cells (BMSCs) by suppressing Wnt signaling and osteogenic markers while inducing osteoblast apoptosis [40,41]. The PI3K/AKT/mTOR and JNK pathways are critical mediators of oxidative stress-induced osteoblast dysfunction [42,43]. Conversely, ROS promote osteoclastogenesis by activating NF-κB and upregulating osteoclast markers such as c-Fos and NFATc1 [44]. The Nrf2/Keap1/ARE pathway counteracts ROS, decreasing bone resorption and oxidative damage [45]. Targeting these pathways with antioxidants offers therapeutic potential [17].

Inflammation, particularly chronic low-grade inflammation in aging, and oxidative stress exert the synergistic effect of exacerbating osteoporosis [46]. ROS amplify pro-inflammatory signaling, while inflammation-induced mitochondrial dysfunction further increases oxidative stress [47,48]. Key cytokines, including TNF-α, IL-1, IL-6, and IL-17, drive osteoclastogenesis by activating the NF-κB and MAPK pathways, increasing RANKL and M-CSF and suppressing OPG [36]. IL-1 and IL-18 further enhance RANKL expression, while TNF-α inhibits Wnt signaling via DKK1 [36]. These findings highlight the dual role of inflammation in directly promoting bone resorption and indirectly amplifying oxidative stress.

Targeting oxidative stress and inflammation represents a promising strategy for osteoporosis treatment. Antioxidants that activate the Nrf2 pathway or reduce ROS production can mitigate bone loss [49]. Similarly, anti-inflammatory agents that inhibit TNF-α, IL-1, or IL-17 signaling may restore bone homeostasis [36]. Combined therapies addressing both pathways could offer synergistic benefits, particularly in aging and postmenopausal osteoporosis.

### 2.3. Cellular Senescence

Cellular senescence, marked by irreversible growth arrest and the senescence-associated secretory phenotype (SASP), is a key driver of aging-related osteoporosis [50]. The SASP, comprising pro-inflammatory cytokines (e.g., IL-6 and IL-8) and immune-modulatory factors, disrupts bone homeostasis by promoting inflammation and impairing remodeling [51]. In the aging-bone microenvironment, senescent osteocytes, myeloid cells, and other bone-resident cells accumulate, exacerbating bone loss through SASP-mediated mechanisms [52,53].

Senescent cells disrupt bone homeostasis by enhancing osteoclast activity and suppressing osteoblast function. The selective elimination of senescent cells or the inhibition of the SASP preserves trabecular and cortical bone mass, highlighting their role in age-related osteoporosis [54]. Aging shifts the differentiation of BMSCs from osteogenic to adipogenic, as evidenced by the reduced expression of osteogenic markers (*Runx2*, Dlx5, collagen, and osteocalcin) and increased adipogenic regulator PPAR-γ2 levels [17]. This imbalance is driven by diminished TGF-β activity and altered BMP signaling, favoring adipogenesis over osteogenesis [55]. Targeting this transition offers a potential therapeutic avenue for osteoporosis.

Mitochondrial dysfunction and impaired mitophagy underpin cellular senescence. Senescent cells exhibit elevated ROS production, accelerating telomere shortening and promoting SASP-driven inflammation [56,57,58]. Sirtuin-3 (Sirt3), a mitochondrial regulator, mitigates BMSC senescence by enhancing mitochondrial function and mitophagy, offering a promising strategy to counteract bone loss [59,60]. Senolytics, SASP inhibitors, and mitochondrial modulators (e.g., Sirt3 activators) represent novel interventions for osteoporosis. These approaches target senescent cells and their inflammatory effects, restoring bone homeostasis in aging populations.

**Figure 2:**
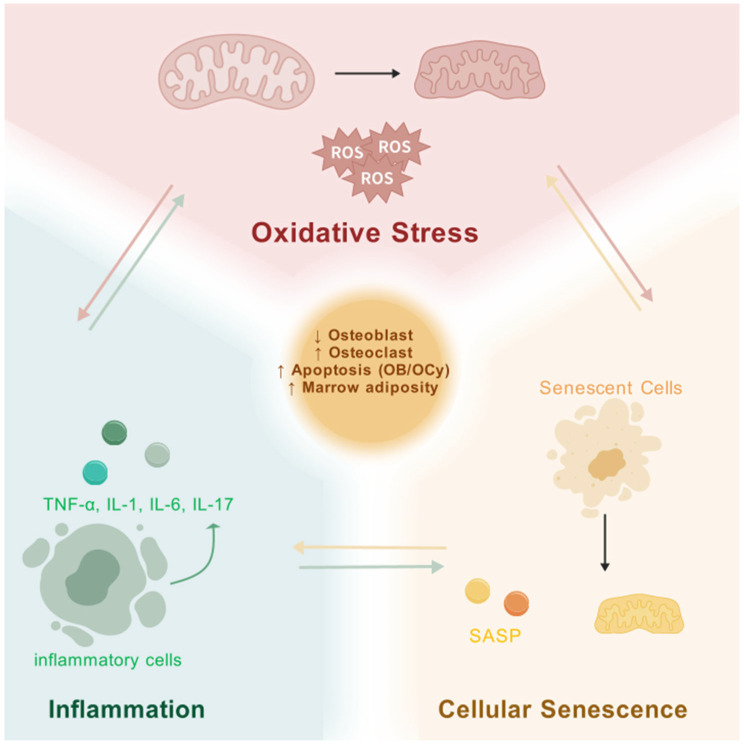
Interplay of Oxidative Stress, Inflammation, and Cellular Senescence in Osteoporosis. These interconnected processes synergistically impair bone homeostasis by enhancing osteoclast activity, suppressing osteoblast function, and promoting bone marrow adiposity. Mitochondrial dysfunction and ROS amplification link all three mechanisms, representing promising therapeutic targets. Created with biogdp.com [33].

We can observe that oxidative stress, inflammation, and senescence interact with each other. Mutually influencing factors are briefly described below (Figure 2).

### 2.4. Osteoimmunology in Osteoporosis: The Interplay Between Bone and Immune Cells

Osteoimmunology explores the intricate crosstalk between bone biology and immunology, emphasizing the role of immune cells in bone remodeling. Lymphocytes, particularly T and B cells, are critical mediators in this process, influencing bone homeostasis through cytokine production and interactions with the RANK/RANKL/OPG axis.

Resting T cells maintain bone mass by supporting OPG production, as evidenced by osteoporosis in T-cell-deficient nude mice [61]. However, activated T cells promote osteoclastogenesis by secreting pro-osteoclastic cytokines such as RANKL and TNF-α. Among the T-cell subsets, Th17 cells are the most potent pro-osteoclastogenic CD4+ cells, producing IL-17A, IL-17F, IL-21, and IL-22 [62]. IL-17 drives osteoclastogenesis by inducing RANK expression [17], while Th17 cells also secrete IL-1, IL-6, TNF, and IFN-γ, further enhancing osteoclast activity [63]. Th17 cells are implicated in various bone diseases, highlighting their pathological significance [62]. Natural killer T (NKT) cells contribute to osteoclastogenesis by producing IFN-γ, which activates macrophages and T cells to secrete TNF-α, and by directly secreting RANKL and M-CSF [64]. In contrast, CD8+ T cells exert a bone-protective role by secreting OPG alongside RANKL [65], while regulatory T (Treg) cells inhibit osteoclastogenesis by suppressing RANKL and M-CSF production, thereby increasing bone volume [66].

B cells play a dual role in bone remodeling. In postmenopausal osteoporosis, B cells exhibit increased surface RANKL expression, contributing to bone loss. Mice lacking RANKL in B cells are partially protected from ovariectomy-induced osteoporosis [67]. Conversely, B-cell knockout (KO) mice develop osteoporosis due to reduced OPG levels, underscoring B cells as a primary source of OPG under physiological conditions [68]. The ability of B cells to regulate the RANKL/OPG balance offers a novel perspective on the pathogenesis of osteoporosis.

Targeting immune cell-mediated pathways, such as Th17 cell activity or the RANK/RANKL/OPG axis, represents a promising strategy for osteoporosis treatment. Modulating Treg cell function or enhancing B-cell-derived OPG production could restore bone homeostasis, particularly in postmenopausal osteoporosis.

### 2.5. Gut Microbiome

The gut microbiome (GM) is a key regulator of bone metabolism, influencing the “microbiota–skeletal” axis through nutrient absorption, immune modulation, and microbial metabolites [69]. Germ-free (GF) mice exhibit increased bone mass, highlighting the GM’s role in bone homeostasis [70]. Interventions such as probiotics and antibiotics can modulate bone mass by altering GM composition [71,72]. The GM enhances the absorption of essential minerals (Ca, Mg, and P) critical to bone mineralization [73]. The microbial fermentation of dietary fiber produces short-chain fatty acids (SCFAs), which improve calcium absorption and promote osteogenic differentiation while inhibiting osteoclast activity [74]. The GM modulates the mucosal immune system, influencing bone remodeling via the GM–immune–bone axis [75]. For example, *Bacillus clausii* enhances Treg cell activity, while SCFAs, particularly butyrate, promote Treg cell generation [76]. Parathyroid hormone (PTH) combined with butyrate induces Treg cells, stimulating CD8+ T cells to produce the osteogenic factor Wnt10b [77]. However, in hyperparathyroidism, PTH expands pro-osteoclastic TNF+ T and Th17 cells, driving bone loss [78]. Targeting the GM through dietary interventions (e.g., prebiotics and probiotics) or microbial metabolites (e.g., SCFAs) offers promising strategies for osteoporosis prevention and treatment.

### 2.6. Epigenetic Regulation

Epigenetics plays a pivotal role in bone homeostasis, regulating osteoblast and osteoclast differentiation through DNA methylation and histone modifications [79]. Aberrant epigenetic changes are increasingly implicated in osteoporosis pathogenesis, offering new avenues for therapeutic intervention.

DNA methylation patterns in osteoporosis patients reveal key insights into bone biology. In whole-blood analyses, differential methylation in genes such as *ABLIM2*, *CDKL5*, *RHOJ*, *PDCD1*, and *ZNF267*, with hypermethylation in *ABLIM2*, *CDKL5*, *RHOJ*, and *PDCD1* and hypomethylation in *ZNF267* were identified [80]. The hypermethylation of *BMP2* downregulates osteoblast markers, while *RUNX2* and *SP7* exhibit reduced methylation during osteoblastic differentiation [80]. Osteogenic lineage-specific genes (*Dlx5*, *Runx2*, *Bglap*, and *Osterix*) are demethylated in osteogenic differentiation, as is osteocalcin (*OCN*), promoting osteogenesis [81,82]. In osteoporotic fractures, lower *RANKL* promoter methylation and higher *OPG* methylation correlate with increased *RANKL* expression and decreased *OPG* expression, which enhance bone resorption [83].

Histone modifications, including acetylation, methylation, phosphorylation, ubiquitylation, and SUMOylation, regulate gene expression in bone metabolism [84]. The inhibition of EHMT2-mediated H3K27 methylation suppresses RANKL-induced osteoclast differentiation [85]. KDM5A reduces H3K4me3 at the *Runx2* promoters, inhibiting BMP2-induced osteogenesis, while Ash1l enriches H3K4me3 at the *Osx*, *Runx2*, *Hoxa10*, and *Sox9* promoters to promote osteogenesis [86]. GCN5 and PCAF enhance osteogenic differentiation by acetylating H3K9 at the Wnt (*Wnt1*, *Wnt6*, *Wnt10a*, and *Wnt10b*) and BMP pathway (*BMP2*, *BMP4*, *SMAD1*, *BMPR1B*, and *RUNX2*) gene promoters, respectively [87,88].

**Figure 3:**
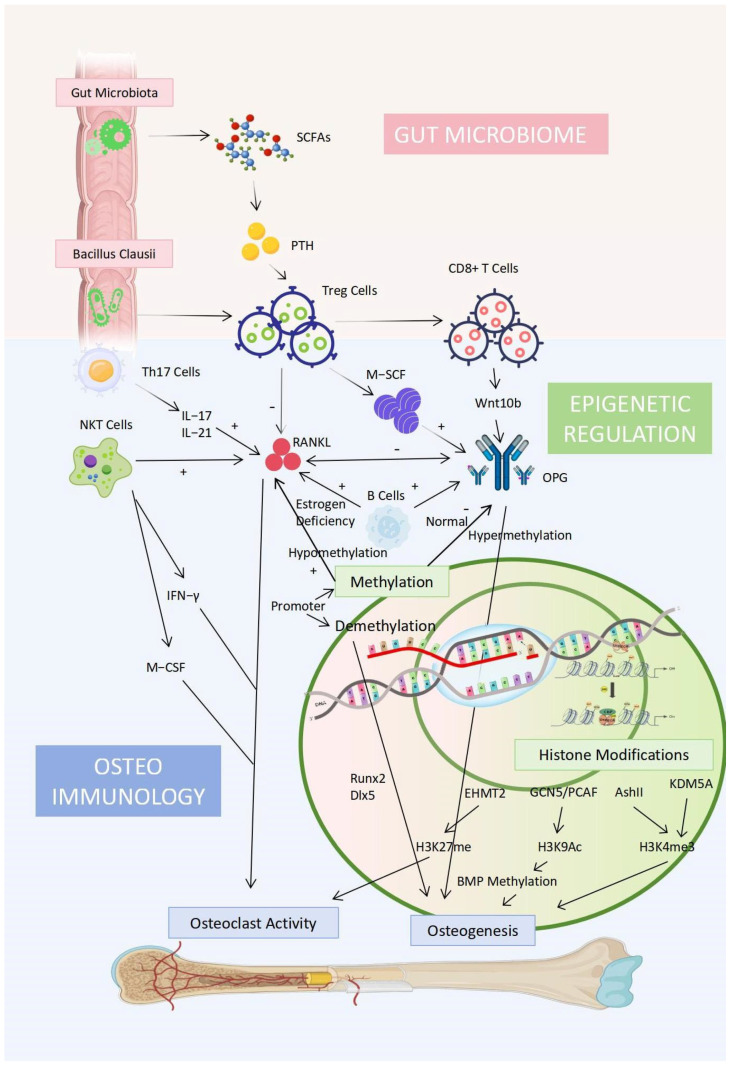
Interplay between osteoimmunology, gut microbiome, and epigenetic regulation in osteoporosis. This diagram summarizes the key mechanisms linking immune responses, gut microbiota, and epigenetic modifications in the regulation of bone metabolism.

In addition, there are also interrelationships among osteoimmunology, the gut microbiome, and epigenetics. The relevant mechanism and connection are presented in Figure 3. Beyond these, osteoporosis is influenced by genetic, nutritional, and lifestyle factors. Genetic factors account for up to 85% of peak bone mass variance, with parental fractures predicting offspring fracture risk [89]. Vitamin D supplementation reduces bone turnover and increases BMD [90]. Gut microbiota-derived short-chain fatty acids (SCFAs), such as butyrate, promote Treg cell generation, which modulates bone metabolism and inflammatory balance [91]. Epigenetic modifications also affect plasma 25-hydroxyvitamin D3 [25(OH)D] levels and cellular senescence in bone cells [92].

## 3. Omics of Osteoporosis

### 3.1. Genomics

Osteoporosis is a multifactorial disease with a strong genetic component, as evidenced by the 60–80% heritability of BMD [93]. GWASs have allowed for the identification of over 1100 loci influencing BMD, though the causal genes for many remain elusive [94]. Key genetic pathways, including the Wnt/β-catenin and RANKL/RANK/OPG signaling cascades, are central to bone homeostasis and remodeling. Understanding these genetic underpinnings offers significant potential for diagnosis, risk stratification, and targeted therapies.

Type I collagen, encoded by *COL1A1* and *COL1A2*, is a major component of the bone matrix. Mutations in these genes, such as the *COL1A1* Sp1 polymorphism (rs1800012), are associated with reduced BMD and increased fracture risk [95]. Rare missense mutations in *COL1A2* (p.Gly496Ala and p.Gly703Ser) have also been linked to osteoporosis [96].

The *LRP5* gene, a coreceptor in the Wnt signaling pathway, is critical to bone formation. Loss-of-function mutations, such as V667M and A1330V, are associated with reduced BMD, vertebral fractures, and early-onset osteoporosis [97,98,99].

The estrogen receptor gene *ESR1* regulates bone mass through estrogen signaling. Polymorphisms such as rs9340799 (XbaI) are associated with BMD variations and fracture risk in postmenopausal women, though findings across populations are inconsistent [100,101].

*SOST* encodes sclerostin, a Wnt pathway inhibitor. SNPs such as rs1513670 and rs7220711 are linked to low-trauma fractures and BMD [102]. The RANKL/RANK/OPG pathway, regulated by *TNFRSF11A* and *TNFRSF11B*, influences osteoclast activity. SNPs in these genes (e.g., rs3018362) are associated with BMD and osteoporosis risk [103]. 

GWASs have not only allowed for the identification of genetic risks but have also facilitated the discovery of therapeutic targets. Five of the eight anti-osteoporosis drugs in clinical use or trials, including denosumab and sclerostin inhibitors, were directly identified with GWASs [104]. Genetic support for drug mechanisms doubles the likelihood of clinical trial success, underscoring the translational potential of genomics [105]. GWASs enable the identification of high-risk individuals and personalized treatment strategies. For instance, genetic loci associated with medication-related osteonecrosis of the jaw (MRONJ) and atypical femoral fractures in bisphosphonate users have been identified, highlighting the utility of genetic screening in mitigating adverse effects [106,107].

### 3.2. Transcriptomics

Transcriptomics, the study of gene expression differences across biological states, has evolved from microarrays to RNA sequencing, offering unprecedented insights into osteoporosis pathogenesis [108]. By analyzing transcriptional profiles, the key genes and regulatory networks involved in bone formation and resorption have been identified, providing novel therapeutic targets and diagnostic biomarkers [93].

Transcriptomic studies highlight the roles of *RUNX2*, *RANKL/OPG*, and *SOST* in bone turnover disorders. For instance, male idiopathic osteoporosis patients exhibit reduced expression of *WNT10B*, *RUNX2*, *RANKL*, and *SOST* in iliac crest biopsies compared with healthy controls [109]. Reduced *RUNX2*, *SP7*, and *SOST* expression has also been observed in osteoporotic femoral neck and head tissues [110]. *RUNX2*, a master transcription factor for osteoblast differentiation, regulates critical signaling pathways, including Wnt, FGF, and hedgehog, to promote osteoblast proliferation and lineage commitment [111]. Similarly, *Sp7* (Osterix), a zinc finger transcription factor, is essential to osteoblast differentiation and bone formation [112]. Conversely, increased *RANKL* expression and elevated *RANKL/OPG* ratios, biomarkers of bone resorption, are associated with osteopenia in postmenopausal women [113].

MicroRNAs (miRNAs) and long non-coding RNAs (lncRNAs) are emerging as critical epigenetic regulators of bone metabolism. miRNAs, such as miR-185, miR-139-5p, and miR-433-3p, modulate osteoblast differentiation by targeting Wnt/β-catenin signaling components (e.g., *CTNNB1* and *FZD4*) [114,115,116]. miR-146a downregulation increases *OPG*, *Wnt2*, and β-catenin expression, inhibiting osteoporosis in ovariectomized rats [117]. Conversely, miR-183 and miR-451a suppress osteoblastogenesis by targeting *Smad4* and *Bmp6*, respectively [118,119].

lncRNAs, transcripts exceeding 200 nucleotides, also regulate bone metabolism. For example, lncRNA *H19* promotes osteoblast differentiation by inhibiting *Dkk4* and activating Wnt signaling [120]. *MSC-AS1* and *MALAT1* enhance osteogenic differentiation, while *CASC11* is upregulated in osteoporosis and promote osteoblast apoptosis or recurrence [121,122,123]. These findings underscore the potential of non-coding RNAs as diagnostic markers and therapeutic targets.

ScRNA-seq provides a high-resolution view of gene expression at the cellular level, revealing unique transcriptional profiles in BMSCs, osteoblasts, and osteoclasts [93]. For instance, *CRIP1* expression is reduced in osteoporosis, and its overexpression rescues bone loss by enhancing osteogenic differentiation [124]. *LRRc17* knockdown ameliorates ovariectomy-induced bone loss, while *STRA6* promotes adipogenic over osteogenic differentiation in osteoporotic MSCs [125,126]. ScRNA-seq has also allowed for the identification of distinct osteoblast subpopulations with unique gene expression profiles, offering new insights into bone cell heterogeneity [127].

Although scRNA-seq can reveal cellular heterogeneity within tissues, it remains unclear how cells interact and organize spatially. Spatial transcriptomics investigates cellular interactions within tissues while preserving their native spatial context [128]. Xue et al. found that SSPCs expressing high levels of platelet-derived growth factor receptor β (PDGFRβ) and Ly6a/Sca-1 are highly enriched within the cortical bone tissue, primarily along the outer periosteal surface. This specific enrichment zone constitutes a critical niche for fracture healing [129]. Using spatial transcriptomic profiling, Jiang et al. demonstrated that the highly metastatic, triple-negative breast cancer cell line MDA-MB-231 disrupts bone homeostasis and inhibits fracture healing within key callus regions, including the hard callus, soft callus, and fibrous interzone [130]. Wang et al. applied scRNA-seq and spatial transcriptomics to primary human femoral head tissue cells. Their analysis revealed that within the inflammatory microenvironment of osteoporosis, macrophages promote osteoclastogenesis through the RETN–Cyclase-Associated Protein 1 (CAP1) complex signaling axis [131]. Furthermore, spatial transcriptomic studies have revealed mechanisms underlying progenitor zonation during embryonic osteochondral development. [132]. Currently, spatial transcriptomics techniques have been predominantly applied to osteoarthritis research; however, their application in osteoporosis studies remains limited and requires further advancement [133].

### 3.3. Proteomics

Proteomics, which has evolved from traditional techniques to high-throughput methods such as mass spectrometry and protein pathway arrays, complements genomics and transcriptomics by providing a comprehensive view of molecular signaling pathways [134]. This approach has allowed for the identification of novel diagnostic and prognostic biomarkers, advancing personalized precision medicine in osteoporosis. In proteomic studies, diverse specimens, including bone tissue, serum/plasma, exosomes, and bone-related cells, are utilized to elucidate disease mechanisms and therapeutic targets.

Bone tissue proteomics offers a direct reflection of osteoporosis pathology. Tandem mass tag (TMT) analysis in ovariectomized (OVX) rats revealed 91 upregulated and 42 downregulated proteins, including increased transferrin receptor (TFR1 and TFRC) and decreased ceruloplasmin (Cp) and BMP-2 [135]. REGγ expression is notably reduced in osteoporotic patients and OVX mouse models [136]. The proteomic profiling of femoral heads from postmenopausal women identified 53 downregulated and 22 upregulated proteins, with GSTP1, LAMP2, COPB1, and RAB5B being implicated in osteoporosis with iron accumulation [137]. Despite its clinical relevance, bone tissue is challenging to obtain and preserve, limiting its widespread use.

Serum and plasma proteomics provide minimally invasive, cost-effective screening options. Common bone turnover markers, such as ALP, procollagen type I N-terminal propeptide (P1NP), and C-terminal telopeptide of type I collagen (CTX), are widely used in clinical practice [8]. The authors of prospective studies have identified 22 serum proteins, including PHLD, SHBG, and APOA1, significantly correlated with BMD [138]. Vitamin D-binding protein (VDBP) levels are inversely associated with BMD [139], while RYR1, APOA1, and FETB are upregulated in osteoporotic postmenopausal women [140]. Novel plasma proteins, such as ASAHL, component C7, and tetranectin, have also been linked to BMD variations [141,142]. Peptide fragments, such as ITIH4, are downregulated in patients with high bone turnover, suggesting their role in osteoclast activity [143].

The proteomic analysis of bone-related cells, though technically challenging, offers insights into cell-targeted therapies. MSCs exhibit upregulated FBLN2 and NPR3 during osteogenic differentiation [144], and high-glucose conditions reduce osteogenesis in MSCs, with annexin A7 and fumarate hydratase having been identified as upregulated proteins [145]. Osteocyte-derived extracellular vesicles (YO-EVs) enriched with tropomyosin-1 (TPM1) enhance matrix stiffness and osteogenesis, highlighting their therapeutic potential for senile osteoporosis [146]. Differential protein expression in circulating monocytes, such as Ras Suppressor-1 (RSU1), superoxide dismutase 2 (SOD2), and glutathione peroxidase-1 (GPX1), contributes to osteoclastogenesis and BMD variation [93]. Annexin A2 (ANXA2) is upregulated in the monocytes of individuals with low BMD and is associated with hip fractures [147,148].

The extracellular bone matrix, comprising collagen and non-collagenous proteins (NCPs), plays a critical role in bone structure and function. Altered posttranslational modifications, such as increased collagen I deamidation, are linked to age-dependent fracture risk [149]. Disrupted glycosylation further compromises bone quality. Proteins such as PLS3, involved in bone mineralization under mechanical stimulation, and AnxA6, critical to extracellular matrix (ECM) mineralization, have emerged as key players in bone homeostasis [150,151]. These findings underscore the potential of bone matrix proteomics in the development of novel therapeutic strategies.

### 3.4. Metabolomics

Metabolomics, the study of the end products of cellular metabolism, provides critical insights into osteoporosis by elucidating disruptions in amino acid, lipid, and energy metabolism [152]. These metabolic changes are closely linked to bone remodeling and have emerged as potential biomarkers and therapeutic targets.

Postmenopausal osteoporosis is associated with disrupted amino acid metabolism. Studies in Japanese women revealed significantly lower levels of glycyl-glycine and cystine, alongside elevated hydroxyproline, in groups with low BMD. Hydroxyproline, a collagen degradation product, serves as a marker of osteoclast-mediated bone resorption [153]. Elevated glutamine and altered levels of taurine, β-alanine, and 5-hydroxycaproic acid have also been identified as potential biomarkers in diverse populations [154,155]. In European-ancestry women, γ-aminobutanoate, threonine, cysteine, taurine, and glutamic acid were significantly associated with BMD [156].

Osteoblasts and osteoclasts require substantial energy for bone remodeling. Estrogen deficiency, as in oophorectomy, increases insulin resistance and disrupts glucose metabolism, leading to elevated glucose and lactate levels [157]. Osteoclast bone resorption is energy-intensive, relying on glycolysis and oxidative phosphorylation for ATP production [158]. Disturbances in the tricarboxylic acid (TCA) cycle, such as reduced citric acid and α-ketoglutaric acid, are observed in osteoporosis and diabetes-related bone disorders [8]. Bone also regulates systemic energy metabolism through hormone secretion, creating a feedback loop between bone and energy homeostasis [159].

Lipid metabolism plays a pivotal role in bone health, with increased bone marrow fat content being associated with bone loss [8]. Elevated triglycerides (TGs) and cholesteryl esters, alongside reduced sphingomyelin, are observed in osteoporotic models [160,161]. Lipidomic studies reveal robust changes in fatty acyls, glycerolipids, and glycerophospholipids in osteoporosis [162]. Oxidized lipids and cholesterol imbalance disrupt bone homeostasis by promoting adipogenesis over osteogenesis via PPARγ signaling and inhibiting Wnt–β-catenin pathways [163]. Targeting lipid regulation offers therapeutic potential for osteoporosis [8].

**Figure 4:**
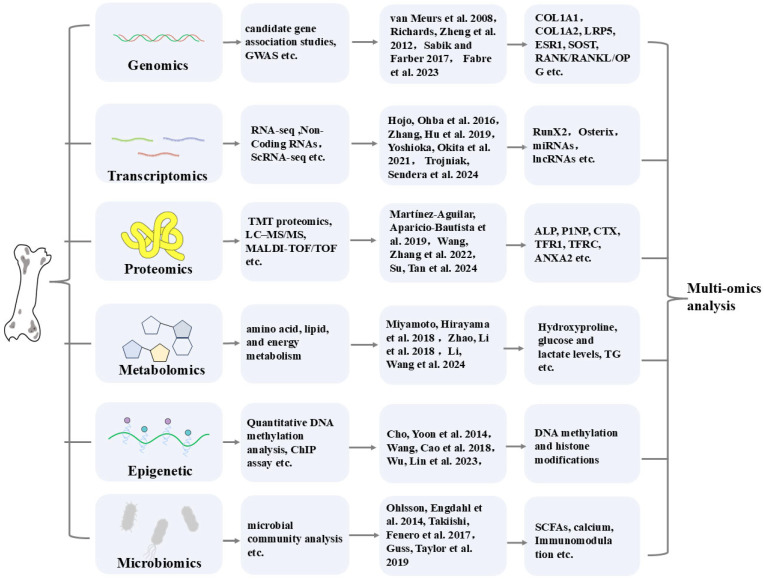
Omics analysis of osteoporosis biomarkers [8,71,72,80,83,96,98,99,104,112,114,121,127,135,137,139,153,162,164,166].

Beyond metabolomics, epigenetic studies highlight the role of DNA methylation in osteoporosis. For instance, Wnt3a-induced osteogenesis is regulated by promoter methylation status [164], and RXRA methylation influences childhood bone mass [165]. The gut microbiome, a key regulator of nutrition, metabolism, and immunity, impacts bone health through short-chain fatty acids (SCFAs), immune modulation, and miRNA regulation [166]. Probiotics and microbiome diversity restoration have shown promise in preventing postmenopausal osteoporosis [167]. The relevant omics research on osteoporosis is presented in Figure 4.

Multi-omics analysis integrates genomic, transcriptomic, methylomic, and metabolomic data to identify biomarkers and biological pathways associated with osteoporosis For example, Qiu et al. identified osteoporosis biomarkers (*FADS2* and *ADRA2A*) and pathways linked to BMD variation [168]. Single-cell and transcriptome analyses revealed neutrophil genes (*DND1* and *HIRA*) as crucial players in osteoporosis development [169]. The authors of a multi-omics analysis of gut microbiota (GM) and metabolites found that the enzymes related to purine and tryptophan metabolism are involved in the progression of OP. They found that these enzymes are from Lachnospiraceae_NK4A136_group, Blautia, Rs-E47_termite_group, UCG-009, and Clostridia_UCG-014 based on gut microbiomics. In a recent study, the authors found that USP6NL, SELENOT, and TAF1A play an important regulatory role in the development of osteoporosis and can be used as potential therapeutic targets through the integration of Mendelian randomization, single-cell RNA sequencing, and bioinformatic analyses [170]. Multi-omics approaches account for genetic, environmental, and lifestyle factors, advancing precision medicine [171].

## 4. Osteoporosis and Comorbidities

Emerging evidence highlights the complex interplay between osteoporosis and chronic conditions such as cardiovascular diseases and immune disorders, mediated by shared pathways including chronic inflammation, oxidative stress, and immunometabolic crosstalk. These interactions not only exacerbate bone fragility but also position osteoporosis as a sentinel indicator of multisystem aging and dysfunction.

### 4.1. Cardiovascular Disease

Epidemiological evidence consistently demonstrates a significant association between osteoporosis and cardiovascular disease (CVD). Studies have shown that postmenopausal women with osteopenia or osteoporosis exhibit higher coronary calcium scores, a marker of atherosclerosis, than controls [172]. Furthermore, lower BMD has been strongly linked to an increased risk of coronary artery stenosis [173]. Elevated homocysteine levels, which negatively correlate with BMD, further underscore this relationship [174]. These findings collectively suggest that osteoporosis and CVD share a bidirectional pathophysiological connection.

The interplay between osteoporosis and CVD is underpinned by shared risk factors, including estrogen deficiency, aging, sedentary lifestyle, and diabetes [173]. At the molecular level, cytokines and signaling pathways such as BMPs, OPG, and Wnt signaling play dual roles in bone metabolism and cardiovascular health. BMPs, known for promoting osteogenic differentiation, also contribute to vascular calcification through pro-inflammatory and pro-oxidative effects, particularly in hyperhomocysteinemia [175]. Similarly, OPG, which inhibits osteoclastic bone resorption, is paradoxically associated with atherosclerosis severity, though it may protect against vascular calcification in certain contexts [176]. Wnt signaling, crucial to bone formation, also exacerbates vascular calcification via the upregulation of Msx-2 and the downregulation of the Wnt inhibitor Dkk1 [177]. Pro-inflammatory cytokines such as IL-6 and TNF-α, which drive bone resorption, are also pivotal to the development of atherosclerosis, highlighting the inflammatory axis as a common pathway [173].

Calcium balance is essential to maintaining bone health, with inadequate intake increasing the risk of osteoporosis and fractures. While dietary calcium positively correlates with BMD and reduces bone loss [178], excessive calcium supplementation has been linked to increased coronary artery calcification (CAC) and elevated cardiovascular mortality [179]. Notably, dietary calcium intake appears safer than supplementation in terms of cardiovascular risk [180]. This dichotomy underscores the need for individualized calcium management in osteoporosis patients.

Pharmacological interventions for osteoporosis also carry cardiovascular implications. Romosozumab, which activates Wnt signaling to enhance bone formation, has been associated with serious cardiovascular adverse events [181]. Conversely, bisphosphonates may reduce atherosclerosis and vascular calcification, though they pose a short-term risk of atrial fibrillation [182]. Thus, the selection of anti-osteoporosis therapies must carefully balance bone health benefits with cardiovascular safety.

### 4.2. Respiratory Diseases

Clinical evidence underscores a significant association between respiratory diseases and osteoporosis, with patients exhibiting a heightened risk of osteopenia and osteoporotic fractures [183]. Chronic obstructive pulmonary disease (COPD), in particular, is strongly linked to osteoporosis, with disease severity correlating with increased bone loss [184]. The pathogenesis of respiratory disease-induced osteoporosis is multifactorial, involving chronic hypoxia and prolonged use of corticosteroids [185].

Dyspnea, a hallmark of respiratory diseases, leads to chronic hypoxia, a key driver of osteoporosis [186]. Hypoxia-induced reactive oxygen species (ROS) accumulation triggers oxidative stress and inflammation, both implicated in bone resorption [187]. Hypoxia-inducible factor-1 alpha (HIF-1α) plays a dual role in this process. On one hand, HIF-1α protects bone cells by inhibiting mitochondrial apoptosis and promoting angiogenesis, essential to bone formation [188]. On the other hand, HIF-1α stimulates osteoclast differentiation via the upregulation of RANKL and NFATc1, exacerbating bone resorption [189]. Thus, maintaining physiological levels of HIF-1α is critical to bone homeostasis.

Corticosteroids, a mainstay in respiratory disease management, significantly impact bone health. While inhaled corticosteroids are effective in controlling symptoms and preventing disease exacerbations, their long-term use has been associated with an increased fracture risk in COPD patients [190]. However, conflicting evidence suggests that fractures may be more closely tied to the underlying disease pathology rather than corticosteroid use itself [191]. Oral corticosteroids, often prescribed for severe respiratory conditions, pose a greater threat, with bone loss being the most pronounced within the first 3–6 months of treatment [192]. These findings highlight the need for careful consideration of corticosteroid regimens to minimize adverse skeletal effects.

Emerging evidence suggests that osteoporosis may conversely exacerbate respiratory disease outcomes. Longitudinal studies have demonstrated that reduced femoral neck bone mineral density (FNBMD) is associated with increased COPD mortality, with men experiencing a higher risk than women [193]. These findings emphasize the bidirectional nature of the relationship, underscoring the importance of addressing bone health in respiratory disease management.

### 4.3. Osteoarthritis

The relationship between osteoarthritis (OA) and osteoporosis (OP) has been a subject of debate. Early studies indicated that OA was associated with higher bone density, with Radin and Rose, in 1986, proposing that OA strengthens subchondral bone, thereby increasing its resistance to deformation [194]. This hypothesis was supported by findings that OA patients tend to develop fragility fractures later than those without OA [195]. However, more recent research has yielded conflicting results regarding the connection between OA and OP. Some studies indicate that higher bone density may reduce OA progression risk [196], while others suggest that genetically predicted low BMD may increase OA susceptibility [197]. In contrast, separate findings suggest that OP might reduce OA incidence and that genetic predisposition to OP negatively correlates with knee OA [196]. A meta-analysis concluded that OA neither protects against OP nor increases its risk beyond that of age- and sex-matched controls, with discrepancies likely due to disease location and progression stage [198,199].

Despite their apparent differences, OA and OP share several pathophysiological mechanisms. Inflammation is a major contributing factor to both conditions. Pro-inflammatory cytokines such as IL-1β, TNF-α, and IL-6 contribute to both OP-related bone loss and OA-associated cartilage degradation [194]. Age-related chronic inflammation (“inflammaging”) further exacerbates these processes by promoting oxidative stress and joint deterioration [200]. Estrogen deficiency is another common factor, as it disrupts cartilage metabolism, accelerates OA progression, and weakens muscle strength, thereby reducing joint stability [201]. At the genetic level, the Wnt signaling pathway plays a role in both OA cartilage development and OP-related bone metabolism [202]. Additionally, gut microbiota-derived inflammatory mediators may contribute to low-grade systemic inflammation, further promoting OA progression [203].

Osteopontin (OPN) plays a pivotal role in both bone and joint health by regulating the activity of chondrocytes, synoviocytes, osteoclasts, and osteoblasts [204]. Elevated OPN levels in plasma, synovial fluid, and cartilage correlate with OA severity and progression [93]. Similarly, OPN is a recognized risk factor for postmenopausal OP and serves as an early diagnostic biomarker for the disease [204]. OPN promotes inflammation and contributes to OA and OP progression by stimulating the production of TNF-α and IL-6, further linking these two conditions [152].

Recognizing the interplay between OA and OP is crucial to optimizing treatment. OA patients require strategies to maintain bone mass and prevent fractures, while OP patients should prioritize joint stability and the early detection of OA symptoms [205]. Beyond conventional approaches such as exercise, calcium, and vitamin D supplementation, several pharmacological interventions show promise for addressing both conditions: Bisphosphonates, widely used for OP, also reduce subchondral bone turnover, mitigate bone loss, and preserve cartilage integrity in OA [206]. Denosumab inhibits NF-κB signaling, reducing chondrocyte apoptosis, subchondral bone remodeling, and cartilage degradation, suggesting its potential role in slowing OA progression [207]. Tocotrienols (TTs), a form of vitamin E, have demonstrated benefits in animal models by improving bone density, reducing oxidative stress and inflammation, and protecting cartilage from degradation [208].

### 4.4. Tumor

The relationship between cancer and osteoporosis has garnered increasing attention in recent years. Cancer and its treatments, particularly chemotherapy, radiation, and hormone therapy, not only affect bone density directly but also exacerbate osteoporosis by altering bone metabolism. In hormone-dependent cancers such as breast cancer, reduced estrogen levels increase bone resorption, contributing to osteoporosis [209]. Chemotherapy and radiotherapy, commonly used to treat cancer, further accelerate osteoporosis by directly damaging bone cells, especially osteoblasts and osteoclasts [210].

Beyond treatment-related effects, certain tumors, particularly non-small-cell lung cancer (NSCLC), can induce osteoporosis by secreting bone resorption factors or osteoclast-promoting signals [211]. For example, elevated osteopontin (OPN) expression in NSCLC patients is linked to the development of bone metastases [212]. Studies indicate that reducing OPN expression may help inhibit osteoclast differentiation [213], suggesting that tumor cells promote osteoporosis by altering bone metabolism and may worsen bone destruction via metastasis.

Bone metastasis often results in osteolytic or osteoblastic lesions, severely impacting patients’ quality of life [214]. This process involves complex interactions between tumor cells and the bone microenvironment, disrupting normal bone remodeling. Verbruggen et al. [215] identified a novel mechanism in breast and prostate cancers whereby osteocytes inhibit tumor cell proliferation through TNF-α secretion. However, tumor cells counteract this inhibition by secreting TGF-β, creating a feedback loop that accelerates metastasis. Such feedback loops are critical to understanding how tumor–osteocyte interactions promote metastasis.

Dumanskiy et al. [216] found that highly metastatic tumors, such as breast and prostate cancer, exacerbate bone metabolism disorders by secreting bone resorption-promoting factors such as OPN and RANKL. Similarly, metabolic reprogramming in the tumor–bone microenvironment has been proposed as a key driver of cancer progression. Whitburn and Edwards [217] reviewed these metabolic adaptations, noting that osteolytic and osteoblastic lesions in breast and prostate cancer are associated with the dysregulation of bone resorption and formation. The altered microenvironment, due to osteoblast and osteoclast metabolism, supports tumor cell survival and growth.

Fan et al. [218] explored osteoclast–cancer cell metabolic crosstalk and its role in resistance to PARP inhibitors in bone metastasis. They found that glutamine metabolism is crucial to cancer cell survival under treatment stress. Moreover, Verbruggen et al. [219] showed that osteocyte paracrine signaling can inhibit tumor cell proliferation and invasion. However, this effect was reversed in breast cancer cells when osteocytes were subjected to mechanical stimulation, emphasizing the role of the mechanical microenvironment in bone metastasis.

The activation of immune cells has also been implicated in bone homeostasis disruption, particularly in cancer patients. In postmenopausal women with low estrogen levels, a low-grade chronic inflammatory state further intensifies bone resorption and accelerates osteoporosis [220].

In summary, the metabolic impact of tumor cells on osteoporosis involves both direct factors from the tumor and the side effects of cancer treatment. Tumor cells promote osteoporosis by secreting cytokines that damage bone cells and alter immune system function. Additionally, hormonal changes, immune cell activation, metabolic reprogramming, and mechanical factors induced by cancer therapies exacerbate bone metabolism disorders, further contributing to the development and progression of osteoporosis.

### 4.5. Neurological Disorders and Their Association with Osteoporosis

The increasing prevalence of neurodegenerative diseases, such as Alzheimer’s disease (AD) and Parkinson’s disease (PD), among the elderly has highlighted their strong correlation with osteoporosis. The coexistence of these conditions poses significant clinical challenges, as neurological disorders can accelerate osteoporosis through multiple mechanisms.

One major contributing factor is the reduced physical activity in patients with AD and PD due to motor dysfunction and cognitive decline, leading to decreased bone strength and increased fracture risk [221]. Beyond immobility, these diseases influence bone metabolism through the brain–bone axis. In AD, the accumulation of amyloid-β and tau protein, key hallmarks of neurodegeneration, has been implicated in osteoporosis development by modulating neuropeptide Y (NPY) expression, which impacts bone density and the trabecular architecture [222]. Similarly, dopamine deficiency in PD disrupts neurotransmitter balance, negatively affecting bone metabolism and increasing fracture susceptibility [223].

Recent studies indicate that brain-derived extracellular vesicles (BEVs) play a crucial role in mediating these effects. BEVs, which carry proteins and RNA, can travel through the circulatory system and influence osteoblast and osteoclast function, ultimately leading to bone loss [224]. Moreover, the sympathetic nervous system is a key regulator of bone homeostasis. Its activation promotes bone resorption by stimulating osteoclast activity while inhibiting osteoblast differentiation. Neuropeptide Y signaling has been identified as a mediator of this process, shifting mesenchymal stem cell differentiation toward adipogenesis at the expense of bone formation [225]. Notably, pharmacological sympathetic blockade has been shown to preserve bone density and improve the trabecular microstructure [226].

Inflammatory pathways further link neurodegeneration and osteoporosis. In AD, elevated levels of pro-inflammatory molecules such as Prokineticin 2 (PROK2) and colony-stimulating factor 3 (CSF3) have been associated with both neuroinflammation and bone loss, suggesting a shared pathological mechanism between these diseases [227].

Conversely, osteoporosis may also influence neurological function via the bone–brain axis. Epidemiological studies indicate that osteoporosis often coexists with cognitive impairment, with fractures accelerating cognitive decline in elderly patients [228]. Bone-derived molecules such as osteocalcin not only regulate bone metabolism but also play essential roles in cognition and memory preservation [229]. Emerging research highlights the potential neuroprotective effects of extracellular vesicles secreted by young osteocytes, which may modulate AD-related pathways and support brain health [230]. Moreover, the dysregulation of the AKT signaling pathway, a key modulator of glucose metabolism, has been implicated in both osteoporosis and neurodegeneration, further reinforcing their interconnected pathophysiology [231].

The management of osteoporosis in patients with neurological disorders presents significant challenges. Many central nervous system medications, including antidepressants, antipsychotics, and antiepileptics, have been shown to adversely affect bone metabolism, exacerbating osteoporosis progression [232]. Conversely, standard osteoporosis treatments, such as bisphosphonates, may influence neurological function, particularly in neurodegenerative conditions [233]. Vitamin D supplementation has demonstrated dual benefits, improving both cognitive function and BMD in these patients, underscoring its potential as a therapeutic intervention [234].

Addressing the bidirectional relationship between neurological disorders and osteoporosis requires an integrated treatment strategy that optimizes bone and brain health while minimizing adverse effects. Future research should be focused on targeted therapies that simultaneously mitigate neurodegeneration and bone loss to improve patient outcomes.

## 5. Conclusions

Osteoporosis is a multifactorial disease, resulting from the combined effects of genetic, metabolic, inflammatory, and environmental factors. Epigenetics and the microbiome also play a role in bone mass regulation. The primary mechanisms involve an osteogenic–osteoclastic imbalance, which disrupts bone metabolism and facilitates the progression of osteoporosis. These etiological factors are interconnected. For instance, inflammation and oxidative stress are significant contributors to senescence and aging phenotypes [235], with aging manifestations including oxidative stress [236,237]. The authors of a previous study mentioned an oxidative stress–inflammation–aging trinity to clarify their interrelationships and highlight their synergistic effect on advancing osteoporosis [238]. In addition, the “microbiota–skeletal” axis can also influence other pathological mechanisms and contribute to bone remodeling, as the gut microbiota can regulate the immune system and produce Treg cells, which have been proven to play a role in bone remodeling. Additionally, PTH can not only act as a hormone to regulate bone development but also work in conjunction with intestinal microbiota to jointly stimulate the production of Treg cells. In recent years, it has been proved that SCFAs, especially butyrate and propionate, could be used as potent histone deacetylases (HDACs) [239], which can repress the function of Runx2 in differentiating osteoblasts [240]. Finally, although not proven to be related to osteoporosis, senescence, and inflammation have been shown to regulate epigenetic modifications. The degree of DNA methylation varies with age, as do the epigenetic modifications of senescent cells [241]. Similarly to the expression of the pro-inflammatory cytokine IL-6, the expression of DNA methyltransferase (DNMT), which is a DNA-modifying enzyme, can be enhanced [242]; DNMT3A upregulation might contribute to SOD2 hypermethylation and increased ROS generation, which may enhance oxidative stress [243].

**Figure 5:**
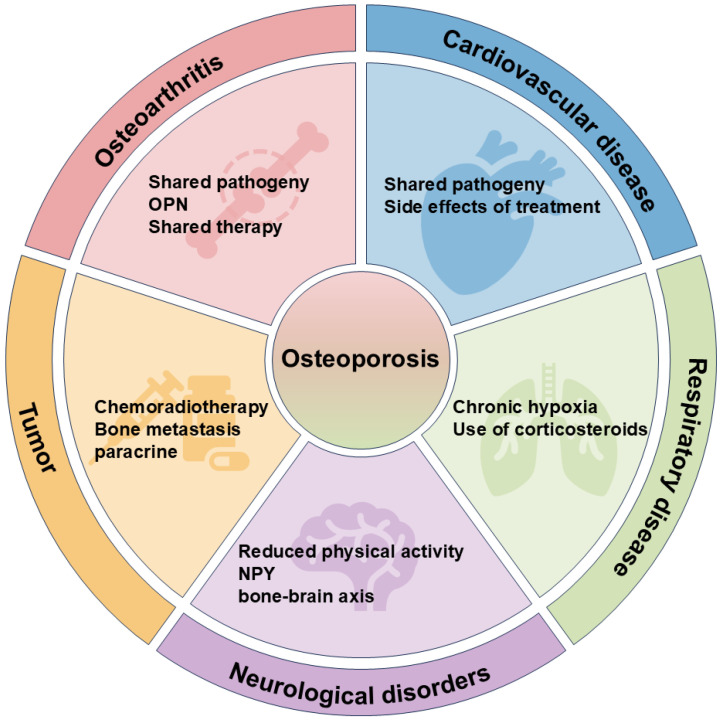
The comorbidity mechanism of osteoporosis with other diseases.

The advent of omics technologies has revolutionized the understanding of osteoporosis, enabling the identification of susceptibility genes and the differentiation patterns of osteoblasts and osteoclasts and diagnostic metabolites and therapeutic targets. Additionally, exploring the comorbidity mechanisms (described in Figure 5) between osteoporosis and other diseases offers novel insights and treatment strategies, while addressing comorbidities helps break the vicious cycle of mutual disease influence. Leveraging omics, particularly emerging technologies, facilitates the development of personalized diagnostic markers and fosters interdisciplinary collaboration across orthopedics, endocrinology, microbiology, and genetics, further advancing osteoporosis research and therapy.

## 6. Future Perspectives

Addressing these multifaceted challenges necessitates focused future efforts. Key priorities include leveraging multi-omics integration and longitudinal studies powered by AI to decipher causal pathways and dynamic interactions over time. Unraveling the precise mechanisms of the microbiome–skeletal axis, resolving the paradoxical roles of metabolites like SCFAs, and exploring targeted epigenetic editing strategies (e.g., CRISPR, DNMT/HDAC modulators) are crucial for understanding and manipulating bone biology. Furthermore, exploiting shared pathological mechanisms with comorbidities (Figure 5), such as chronic inflammation, offers novel therapeutic avenues to disrupt disease cycles. The ultimate translation lies in precision medicine: developing integrated diagnostic panels (genetic, epigenetic, microbiome, metabolic) for early risk stratification and personalized interventions, alongside biomarkers predicting treatment response. Overcoming these challenges through robust interdisciplinary collaboration is paramount to harnessing converging insights and advancing toward predictive, preventive, and personalized osteoporosis management.
